# MicroRNA-762 Is Upregulated in Human Corneal Epithelial Cells in Response to Tear Fluid and *Pseudomonas aeruginosa* Antigens and Negatively Regulates the Expression of Host Defense Genes Encoding RNase7 and ST2

**DOI:** 10.1371/journal.pone.0057850

**Published:** 2013-02-28

**Authors:** James Mun, Connie Tam, Gary Chan, Jong Hun Kim, David Evans, Suzanne Fleiszig

**Affiliations:** 1 School of Optometry, University of California, Berkeley, California, United States of America; 2 Vision Science Program, University of California, Berkeley, California, United States of America; 3 Program in Bioengineering, University of California, Berkeley, California, United States of America; 4 Program in Molecular and Cell Biology, University of California, Berkeley, California, United States of America; 5 College of Pharmacy, Touro University California, Vallejo, California, United States of America; 6 Graduate Groups in Microbiology and Infectious Disease, University of California, Berkeley, California, United States of America; University of California Merced, United States

## Abstract

Mucosal surfaces regulate defenses against infection and excessive inflammation. We previously showed that human tears upregulated epithelial expression of genes encoding RNase7 and ST2, which inhibited *Pseudomonas aeruginosa* invasion of human corneal epithelial cells. Here, microRNA microarrays were used to show that a combination of tear fluid exposure (16 h) then *P. aeruginosa* antigens (3 h) upregulated miR-762 and miR-1207, and down-regulated miR-92 and let-7b (all > 2-fold) in human corneal epithelial cells compared to *P. aeruginosa* antigens alone. RT-PCR confirmed miR-762 upregulation ∼ 3-fold in tear-antigen exposed cells. Without tears or antigens, an antagomir reduced miR-762 expression relative to scrambled controls by ∼50%, increased expression of genes encoding RNase7 (∼80 %), ST2 (∼58%) and Rab5a (∼75%), without affecting *P. aeruginosa* internalization. However, *P. aeruginosa* invasion was increased > 3-fold by a miR-762 mimic which reduced RNase7 and ST2 gene expression. Tear fluid alone also induced miR-762 expression ∼ 4-fold, which was reduced by the miR-762 antagomir. Combination of tear fluid and miR-762 antagomir increased RNase7 and ST2 gene expression. These data show that mucosal fluids, such as tears, can modulate epithelial microRNA expression to regulate innate defense genes, and that miR-762 negatively regulates RNase7, ST2 and Rab5a genes. Since RNase7 and ST2 inhibit *P. aeruginosa* internalization, and are upregulated by tear fluid, other tear-induced mechanisms must counteract inhibitory effects of miR-762 to regulate resistance to bacteria. These data also suggest a complex relationship between tear induction of miR-762, its modulation of innate defense genes, and *P. aeruginosa* internalization.

## Introduction

Many mucosal epithelia in humans and animals have evolved to co-exist with a rich and complex microbiota, but have also retained the ability to respond to potential pathogens, and regulate inflammatory responses to avoid tissue damage and disease [1]–[4]. All of these mucosal epithelial surfaces are bathed in fluids, and yet little is known about how these fluids influence epithelial regulation of innate defense and inflammation.

Like many other tissues surfaces, e.g. airways, gastrointestinal and urogenital tracts, the ocular surface is bathed in a surface liquid, the tear film. We have shown that tear fluid protects ocular surface epithelial cells against bacterial virulence mechanisms *in vitro* [5], [6] and *in vivo* [6], and that *in vitro* protection can be independent of direct antimicrobial activity [5], [7], [8]. Indeed, we recently showed that tear fluid can directly stimulate epithelial cells *in vitro* to enhance their resistance to bacterial virulence mechanisms. Correspondingly, tear fluid enhanced activation of NFκB and AP-1 transcription factors in response to bacterial antigens, and upregulated epithelial-derived innate defense genes. The latter included genes encoding RNase7 and ST2, both of which reduced bacterial internalization by corneal epithelial cells [9]. RNase7 is a potent antimicrobial peptide, active against a broad range of bacterial pathogens, that was originally identified from the *stratum corneum* of human skin [10]. Keratinocytes are the major source of this secreted innate defense protein in human skin [10]. RNase7 has been shown to be present in other epithelia in different organ systems including the respiratory, urinary and gastrointestinal tracts, and the eye (cornea) [10]–[13]. While RNase7 is constitutively expressed in these tissues, it can also be upregulated in response to various stimuli including proinflammatory cytokines (e.g. IL-1β) and microbial antigens [9], [10], [13]. ST2, on the other hand, has been shown to have an immunomodulatory role in innate defense, e.g. negative regulation of IL-1 receptor and TLR-4 receptor signaling [14], [15]. ST2 is constitutively expressed in the corneal epithelium, and its immunomodulatory role is important for resolution of *P. aeruginosa* corneal infections in murine models by promoting Th2-mediated immune responses [16], [17].

The aim of the present study was to further elucidate the mechanisms by which tear fluid modulates epithelial cell susceptibility to *P. aeruginosa* internalization, and the relationship to RNase7 and ST2 gene expression. Since epithelial cells become exposed to tear fluid when they reach the ocular surface through a process of exfoliation, the induction of resistance to microbes would need to be rapid. The hypothesis tested was that tear fluid effects on epithelial cells involve the induction of microRNA expression to modify innate defense gene responses to bacterial challenge.

MicroRNAs are small, 20- to 24 nucleotide, noncoding RNAs found in diverse organisms, which bind partially to the 3’ UTR of their target mRNA to post-transcriptionally silence the target gene [18], [19]. These endogenous, silencing RNAs play important roles in cell and tissue development and differentiation [20], [21], cell signaling and migration [22], [23], cellular stress responses [24], and resistance to bacterial virulence *via* gene suppression [25]. Moreover, microRNAs have been shown to play a role in modulating expression of innate defense genes including Toll-like receptors, their adaptor proteins, downstream signaling pathways, and transcription factors [26].

Since numerous microRNAs are expressed at the ocular surface [27], microarray analysis was performed to determine which microRNAs (miRs) were differentially regulated in corneal epithelial cells by bacterial antigens with and without tear fluid exposure. The results showed a selective and specific up- and down-regulation of four types of miR in tear fluid treated cells, of which miR-762 showed the greatest upregulation. A combination of antagomir [28] and microRNA mimic was then used to show that tear-induced miR-762 negatively regulates RNase7 and ST2 gene expression in corneal epithelial cells. Since expression of these two factors, which inhibit bacterial internalization, is also upregulated by tears, the data suggest that other tear-induced mechanisms must antagonize the inhibitory effects of miR-762 in regulating epithelial resistance to bacterial challenge.

## Materials and Methods

### Ethics statement

Reflex tear fluid was collected from the lower conjunctival sac of healthy human volunteers. Informed written consent was obtained from all participating subjects. These procedures were approved by the Committee for the Protection of Human Subjects (CPHS), University of California, Berkeley (protocol CPHS#2010-09-2111).

### Cell culture

Human telomerase-immortalized corneal epithelial cells (hTCEpi) were maintained in 10 cm tissue culture treated Petri dishes (Becton Dickinson, Franklin Lake, NJ) in serum-free KGM-2 medium (Lonza, Walkersville, MD) until confluent as previously described [29]. Cells were then seeded onto 96-well tissue culture plates (Becton Dickinson) and grown to ∼ 80–90% confluence. At 2 days prior to experiments, cells were incubated in high calcium KGM-2 (i.e. serum-free KGM-2 supplemented with 1 mM Ca^2+^) for 16 h, then washed with sterile phosphate-buffered saline (PBS; Sigma, St. Louis, MO) before exposure to 40 µl of either fresh undiluted human tear fluid or high calcium KGM-2 without antibiotics for either 6 or 16 h depending on the experiment. Cells were incubated at 37°C, 5 % CO_2_, in culture and during experiments.

### Tear fluid collection

Reflex tear fluid was collected from the lower conjunctival sac of healthy human volunteers who had not worn contact lenses for at least three days using microcapillary tubes, as previously described [6]. Tears from 3 different subjects were pooled and stored at -80°C in aliquots.

### Preparation of bacteria and bacterial antigens

*P. aeruginosa* clinical isolate 6294 was used for invasion assays [5]. All bacteria were grown on trypticase soy agar (TSA) plates overnight at 37°C. Bacteria were suspended in basal cell culture media (i.e. serum-free KGM-2 without growth factor “bullet” kit) to a spectrophotometer optical density of ∼0.1 at OD_650_ equivalent to ∼1×10^8^ CFU (colony-forming units)/ml, and diluted to ∼1×10^6^ CFU/ml for invasion assays. Bacterial antigens of *P. aeruginosa* strain PAO1 were prepared as previously described [30]. Briefly bacteria were grown as single colonies on TSA at 37°C overnight. A single colony was then grown at 37°C overnight with aeration to late-logarithmic phase in 5 ml of trypticase soy broth (TSB). The culture broth was centrifuged at 14,000 rpm (16,000 x g) for 30 min and the supernatant collected in a syringe, sterilized with a 0.22 µm polymer filter (Corning Star Corporation, Cambridge, MA), aliquoted and stored at -80°C until use. Bacterial supernatant (bacterial antigens) was diluted 1∶5 with basal cell culture media to stimulate the corneal epithelial cells for experiments.

### RNA purification

Total RNA of hTCEpi incubated under different conditions (e.g. pre-exposed to media or tear fluid for 16 h followed by 3 h incubation with *P. aeruginosa* antigens; transfected with microRNA antagomirs or mimics; exposed to cell culture media or human tear fluid for 6 h) was extracted using the RNeasy Kit (Qiagen, Valencia, CA) with Qiashredder columns used for cell lysis and inserting Qiagen on-column DNase steps to remove contaminating genomic DNA.

### RNA target preparation/GeneChip microarray analysis

RNA quality was assessed using RNA pico & small RNA chips on a 2100 Bioanalyzer (Agilent Technologies, Palo Alto, CA). 100 ng of total RNA was processed for use on the microarray using the FlashTag™ HSR labeling kit (Genisphere LLC, Hatfield, PA) according to manufacturer's protocols. The resultant biotinylated cRNA was hybridized to the GeneChip miRNA Array (Affymetrix). The arrays were washed, stained, and scanned using the Affymetrix Model 450 Fluidics Station and Affymetrix Model 3000 scanner using the manufacturer's recommended protocols by the University of California Functional Genomics Lab. Expression values were generated using miRNA QC tool software (Affymetrix). Evaluation and normalization of Affymetrix genechip data .Cel files were subjected to the Affymetrix miRNA QC Tool. Expression levels were analyzed on a logarithmic scale. Level changes were determined by dividing the experimental group (tear fluid with bacterial antigens) by the control (media with bacterial antigens). A gene showing differential expression by at least two fold was considered for further study.

### Transfection with microRNA antagomir and mimic

The hTCEpi were transfected with scrambled control antagomir/mimic (100 nmol/L) or miR-762 antagomir/mimic using Lipofectamine RNAi Max (Invitrogen, Carlsbad, CA) for 6 h. Cells were used for assays at 48 h after transfection.

### Real-Time PCR

1 µg of total purified RNA was converted to cDNA using a RETROscript Kit (Ambion, Austin, TX) to detect mRNA expression levels of RNase7, ST2, Rab5a, hBD-2, and hBD-3 using CFX96 Real-Time PCR Detection System (Bio-Rad, Hercules, CA). Results were normalized to GAPDH. For miR-762, 1 µg of total purified RNA was converted to cDNA using miScript Reverse Transcriptase Mix (Qiagen, Valencia, CA) and miR-762 level determined by real-time PCR and normalized to U6. MiR-762 antagomirs, mimics and primers were designed by Qiagen (Valencia, CA).

### Invasion assays

The hTCEpi were either pre-exposed to high Ca^2+^ media or human tear fluid for 16 h (for effect of tear fluid pre-exposure experiments), washed with 100 µl of pre-warmed PBS, and 40 µl of 6294 diluted in cell culture media or tear fluid to a concentration of ∼1×10^6^ CFU/ml was inoculated onto each well (in triplicates) for 3 h at 37°C, 5 % CO_2_. The cells were then washed with PBS and incubated with 100 µl of gentamicin (0.4% [vol/vol]; 200 µg/ml; BioWhittaker, Walkersville, MD). After 1 h incubation at 37°C, the cells were lysed in Triton X-100 (0.25% [vol/vol]; LabChem Inc., Pittsburgh, PA) in PBS for 15 minutes. Cells were scraped for complete lysis and bacteria enumerated by viable count using MacConkey agar.

### Statistical analysis

Data were expressed as a mean + SEM (or a mean + SD for invasion assays) (n  =  3 or 4 samples per group) unless otherwise stated. Student’s t-Test was used to evaluate the statistical significance of differences between two groups, e.g. miR antagomir (or mimic) versus scrambled control. P < 0.05 was considered significant. Experiments were repeated at least three times.

## Results

### Tear fluid modulates specific corneal epithelial microRNA responses to bacterial antigens

**Figure 1:**
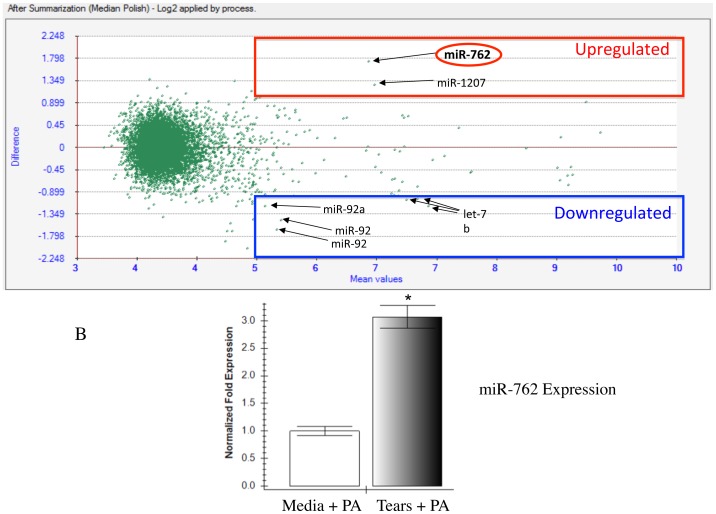
Upregulation of miR-762 in human corneal epithelial cells in response to human tear fluid and *P. aeruginosa* antigens. a Microarray analysis of human corneal epithelial microRNA expression in response to *P. aeruginosa* antigens (3 h incubation) with and without prior exposure to human tear fluid for 16 h (see Material and Methods). Red and blue boxes indicate microRNAs which were up- or down-regulated respectively by 2-fold or more in response to bacterial antigens with prior tear fluid exposure (expressed relative to bacterial antigen challenge without prior tear exposure). The y-axis is shown as log2 (comparing tear fluid + antigens versus media + antigens) with a value of 1 or -1 meaning that the test group was up- or down-regulated respectively by 2 fold. The x-axis shows mean values of the test and control and represent raw data values from the microarray. Only values greater than 5 were considered. Those below 5 were below detection (i.e. considered background). Since there were multiple data points between 3–4 and 6–7 the software generated the figure to distribute these data points along the x-axis. PA  =  *P. aeruginosa* antigens, Media  =  High Calcium KGM-2. Eight wells of hTCEpi (grown in 96-well plates) were pooled to obtain sufficient RNA for each treatment group, b Real-time PCR confirmed a ∼3-fold upregulation of miR-762 in tear-treated epithelial cells in response to antigenic challenge (* Significant difference vs. control, t-Test).

Microarray analysis was used to compare microRNA expression in human corneal epithelial cells after 16 h exposure to undiluted human tear fluid or cell culture media (control), and 3 h exposure to bacterial antigens (see Methods). Very few corneal epithelial microRNAs were up or down-regulated (2 and 6 probe sets, respectively) by 2-fold or more in tear and bacterial antigen treated cells relative to controls (Fig. 1 a). MiR-762 and miR-1207 were both significantly upregulated; miR-92 and let-7b were both significantly downregulated. MiR-762 showed greatest upregulation in tear and bacterial antigen treated cells, which was confirmed by RT-PCR (> 3-fold, Fig. 1 b, p < 0.05, t-Test). These microarray data have been deposited in NCBI's Gene Expression Omnibus [31], and are accessible through GEO Series accession number GSE39341. ().

### An Antagomir to miR-762 enhances mRNA expression of Rab5a, RNase7, and ST-2, but does not influence bacterial internalization

**Figure 2:**
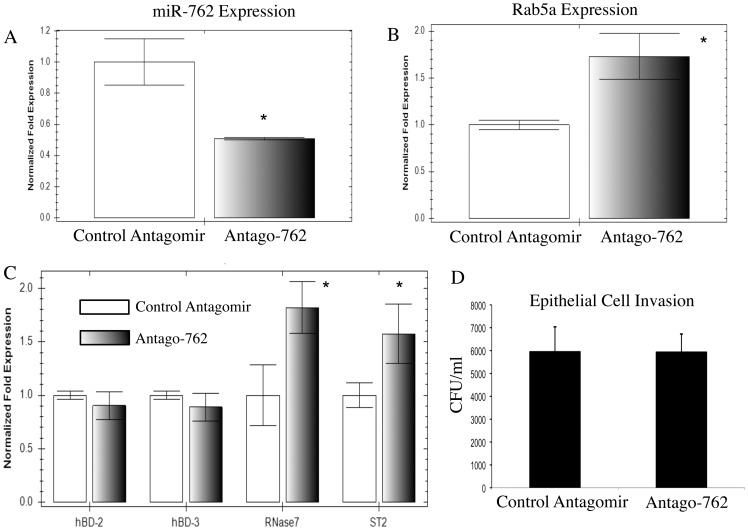
The effect of a miR-762 antagomir on gene expression in human corneal epithelial cells and on *P. aeruginosa* invasion. a Transfection of human corneal epithelial cells with an antagomir of miR-762 (antago-762) decreased the expression of miR-762 relative to a control (scrambled) antagomir, and b increased the expression of Rab5a mRNA, a predicted target of miR-762. c Antago-762 also increased the expression of RNase7 and ST-2 mRNA suggesting that miR-762 negatively regulates these innate defense genes. Antago-762 did not affect genes encoding hBD-2 and hBD-3. d The antagomir did not affect epithelial susceptibility to *P. aeruginosa* invasion. Gene expression was measured by real-time PCR, and *P. aeruginosa* invasion by using gentamicin exclusion assays. In each experiment, cells were used at 48 h after transfection. For invasion assays, epithelia were challenged at 72 h with 10^4^ cfu invasive *P. aeruginosa* strain 6294 for 3 h followed by 1 h gentamicin treatment (see Methods). (* Significant difference vs. respective controls, t-Test).

We have previously shown that tear fluid upregulates epithelial genes encoding RNase7 and ST-2, and that these innate defense factors can protect epithelial cells against bacterial internalization [9]. Since miR-762 was the most profoundly upregulated microRNA by bacterial antigens in tear fluid treated cells (Fig. 1), we tested if miR-762 could influence epithelial expression of genes encoding RNase7 and/or ST-2, and/or affect bacterial internalization. Corneal epithelial cells were transfected with an antagomir to miR-762 (Antago-762) for 48 h under baseline conditions, i.e. without tear fluid or bacterial antigen exposure, to reduce miR-762 expression, and in turn, affect mRNA levels of genes targeted by this microRNA. As expected, Antago-762 effectively reduced epithelial expression of miR-762 at 48 h by ∼50% relative to a scrambled control (Fig. 2 a, p < 0.05, t-Test). Accordingly, Antago-762 increased expression of Rab5a mRNA, a predicted target of miR-762 (based upon analysis of predicted targets for miR-762 using microRNA.org), by ∼75% relative to the scrambled control (Fig 2 b, p < 0.05, t-Test). Interestingly, Antago-762 also increased gene expression of RNase7 (by ∼80%) and ST-2 (by ∼58%) (Fig. 2 c, p < 0.05, t-Test for each), suggesting that miR-762 negatively regulates the expression of genes encoding these innate defense factors. Antago-762 did not affect the expression of genes encoding hBD-2 and hBD-3 (Fig. 2 c), two other antimicrobial peptides expressed by these epithelia [32], [33], nor did it affect bacterial internalization by these epithelial cells (Fig. 2 d).

### A mimic of miR-762 reduces expression of genes encoding RNase7 and ST-2, and increases epithelial susceptibility to bacterial invasion

**Figure 3:**
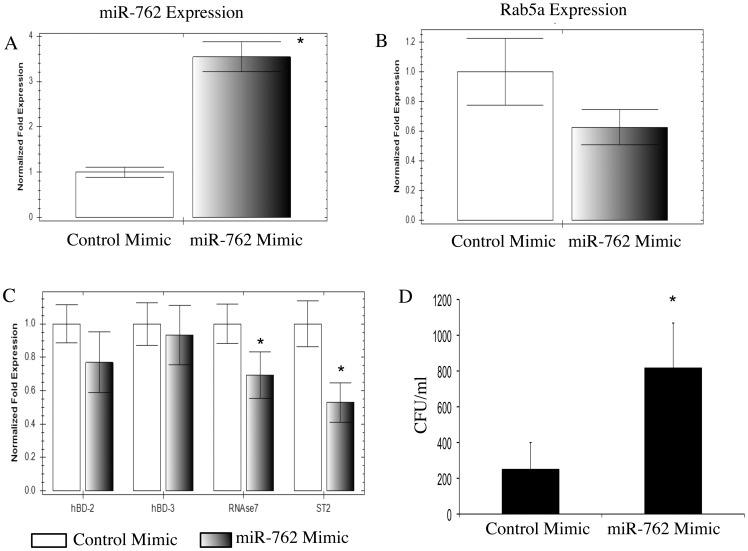
The effect of a miR-762 mimic on gene expression in human corneal epithelial cells and on *P. aeruginosa* invasion. a Transfection of human corneal epithelial cells with a mimic of miR-762 increased the expression of miR-762 relative to a control (scrambled) mimic, **b** decreased Rab5a mRNA expression (although that difference was not significant), **c** significantly decreased mRNA expression of RNase7 and ST-2, but not that of hBD-2 or hBD-3, and **d** increased *P. aeruginosa* invasion (* p  =  0.044, t-Test). Gene expression was measured by real-time PCR, and *P. aeruginosa* invasion using gentamicin exclusion assays. In each experiment, cells were used at 48 h after transfection. At 72 h, epithelia were challenged with 10^4^ cfu *P. aeruginosa* strain 6294 for 3 h followed by 1 h gentamicin treatment (see Methods). (* Significant difference vs. respective controls, t-Test).

Transfection with an antagomir of miR-762 did not reduce bacterial internalization, even though the suppression of miR-762 increased RNase7 and ST-2 mRNA levels. Therefore, we over-expressed miR-762 by transfection with a miR-762 mimic, and compared mRNA levels of target genes and bacterial internalization compared to a scrambled control. Real-time PCR confirmed successful transfection of miR-762 after 48 h (Fig. 3 a). Over-expression of miR-762 suppressed Rab5a mRNA expression by ∼35% although this was not statistically significant (Fig. 3 b, p > 0.05, t-Test). However, miR-762 suppression of RNase7 (by ∼30%) and ST-2 (by ∼47%) mRNA expression was significant (Fig. 3 c, p < 0.05, t-Test, for each versus respective controls). The miR-762 mimic did not significantly affect mRNA levels of hBD-2 or hBD-3 (Fig. 3 c). The miR-762 mimic increased susceptibility to bacterial internalization by ∼3.5-fold (Fig. 3 d, p < 0.05, t-Test).

### Tear fluid induction of miR-762 negatively regulates RNase7 and ST2

**Figure 4:**
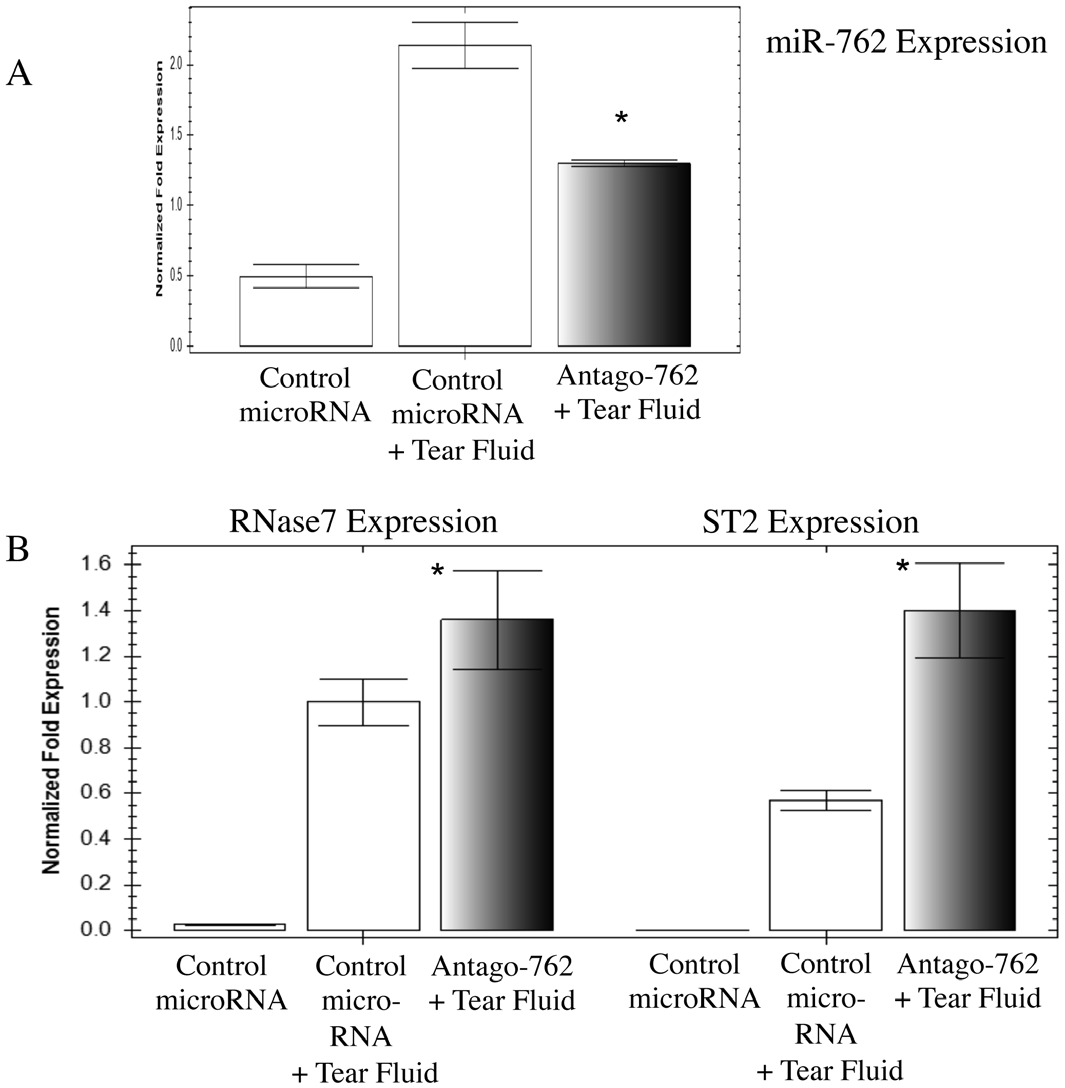
Tear fluid induction of miR-762 negatively regulates RNase7 and ST2 gene expression. a Tear fluid induction of miR-762 expression in corneal epithelial cells was reduced by the antagomir of miR-762. **b** The antagomir of miR-762 enhanced tear-induced expression of RNase7 and ST2 mRNA compared to control (scrambled) antagomir suggesting that tear-induced miR-762 expression negatively regulates these innate defense genes. RT-PCR was used to measure gene expression at 48 h after transfection (see Methods). (* p < 0.05, t-Test versus tear-treated scrambled controls).

Having shown tear-antigen upregulation of miR-762, and miR-762 negative regulation of genes encoding RNase7 and ST2 without tears, we next used a miR-762 antagomir to test the relationship between tear exposure, miR-762 induction, and RNase7 and ST2 mRNA expression. Corneal epithelial cells were transfected with Antago-762 or an irrelevant antagomir (control), then exposed to cell culture media or tear fluid for 16 h and tested for the expression of endogenous miR-762. Tear fluid alone upregulated the expression of miR-762 (∼ 4-fold), which was partially reduced by the miR-762 antagomir (Fig. 4 a). The reduction in tear-induced miR-762 upregulation in antagomir treated cells versus scrambled controls was significant (p < 0.05, t-Test). In the presence of antagomir, tear fluid induced an even greater expression of RNase7 or ST2 mRNA than that found in the presence of scrambled controls (Fig. 4 b, p < 0.05, t-Test, for each comparison) confirming that miR-762 serves to negatively regulate the expression of these tear-induced innate defense genes.

## Discussion

The results of this study show that mucosal fluid can influence epithelial microRNA expression to regulate expression of innate defense genes. Using a model of corneal epithelial cells exposed to natural human tear fluid, we showed that; 1) tear fluid treatment followed by bacterial antigens upregulates miR-762 and miR-1207, and down-regulates miR-92 and let-7b compared to bacterial antigens alone, 2) miR-762 negatively regulates the expression of genes encoding the antimicrobial RNase7, the immunomodulator ST2, and the RhoGTP-binding protein Rab5a, but not the defensins hBD-2 or hBD-3, 3) over-expression of miR-762 suppresses RNase7 and ST2 mRNA levels, and increases bacterial internalization, and 4) tear fluid alone induces miR-762 expression, which negatively regulates genes encoding RNase7 and ST2. Since both RNase7 and ST2 can protect against bacterial invasion [9], and like miR-762, are upregulated by tear fluid, these data suggest that tear induction of miR-762 helps counter over-expression of specific innate immune factors, and that other tear-induced factors antagonize or counteract miR-762 in regulating epithelial cell defenses against bacterial challenge.

This study focused on miR-762, the microRNA that showed the greatest change (either up- or down-regulated) in the microarray analysis (> 3-fold upregulation when verified by RT-PCR), and which was upregulated ∼ 4-fold by tear fluid alone. While little is known about miR-762, it is upregulated in animal models of diabetic nephropathy [34] and oral carcinoma [35]. MiR-762 has been found ubiquitously distributed in murine ocular tissues [36]. Its capacity to negatively regulate at least two different types of innate defense gene, RNase7 and ST2, suggests a role in regulation of ocular innate defense in response to infection that might also occur at other mucosal surfaces. That role would be consistent with previously documented roles for other microRNAs in this regard, e.g. the modulation of TLR responses from the level of receptors, to downstream signaling, to cytokine expression [26], [37]. Further studies are needed to determine the how tear fluid upregulates miR-762, the influence of microbial antigens in that regard, and if negative regulation of RNase7 and ST2 genes involves direct effects or an intermediate factor(s). The latter possibility may be more likely since analysis using microRNA.org did not predict that RNase7 or ST2 would be direct targets of miR-762. The significance of miR-762 in influencing the pathophysiology of ocular (or other) epithelial diseases involving altered exposure to tear (or mucosal) fluid also warrants further investigation.

Suppression of RNase7 and ST2 expression, by over-expressing miR-762 with a microRNA mimic, corresponded with increased bacterial internalization (> 3-fold). This observation was consistent with our previous findings that tear fluid induces the expression of genes encoding RNase7 and ST2, and siRNA knockdown of those genes increases epithelial cell internalization by *Pseudomonas aeruginosa* [9]. However, in the present study, reduced bacterial internalization did not occur when expression of RNase7 and ST2 was enhanced with a miR-762 antagomir. This might relate to the fact that antagonizing miR-762 also enhanced expression of Rab5a, known to be important for mammalian cell endocytosis and intracellular trafficking [38]. Moreover, it is very likely that miR-762 regulates the expression of numerous other genes some of which might also impact bacterial internalization. Whatever is the explanation for these internalization results, the data suggest that tear-induction of miR-762 does not mediate tear suppression of bacterial internalization, and that other tear-induced corneal epithelial factors (microRNA or otherwise) are responsible for that previously reported observation [9].

Epithelial surfaces play a vital role in innate defense against microbial pathogens, and in many instances, allowing mucosal colonization by commensal microflora. MicroRNAs have emerged as important contributors to the regulation of epithelial innate immunity. This study, and our previous study [9], support the hypothesis that tear fluid is important in regulating ocular innate defenses, and that microRNA induction or repression is involved.
